# The Role of a Primary Arthroplasty in the Treatment of Proximal Tibia Fractures in Orthogeriatric Patients

**DOI:** 10.1155/2016/6047876

**Published:** 2016-01-18

**Authors:** Thomas Haufe, Stefan Förch, Peter Müller, Johannes Plath, Edgar Mayr

**Affiliations:** Department of Traumatology, Klinikum Augsburg, Stenglinstraße 2, 86156 Augsburg, Germany

## Abstract

The total knee arthroplasty (TKA) is the gold standard for patients with an advanced symptomatic gonarthrosis. However, there are very few publications dealing with the primary TKA for patients with a proximal tibia fracture. In our retrospective study we evaluated 30 patients treated with a TKA for a proximal tibia fracture in our institution between 01/2008 and 12/2014. We collected the following statistical data from each patient: age, classification of the fracture (AO-classification), type of prosthesis used, length of the operation and hospitalization, and complications during the follow-up. We used the Knee Society Score (KSS) and the WOMAC score to evaluate the function. The Knee Society Score showed an average “general knee score” (KSS1) of 81.1 points and an average “functional knee score” (KSS2) of 74.5 points. The average WOMAC score was 78.6 points. Immediate postoperative mobilization with the possibility of a full-weight bearing is of crucial importance for the geriatric patients to maintain the mobility they had prior to the operation and reduce medical complications. Because of these advantages, the primary TKA seems to be a promising alternative to the ORIF of a proximal tibia fracture in the orthogeriatric patient.

## 1. Introduction

The total knee arthroplasty is the gold standard for patients with an advanced, symptomatic gonarthrosis. It is a well-established therapy based on numerous studies [1–3]. In contrast there are not many publications dealing with the primary arthroplasty for complex tibia plateau fractures. In the current literature there are only a few studies, each with a very small patient population of less than 15, dealing with this subject (Table 1). However the TKA for a proximal tibia fracture seems to be a promising alternative, especially for the increasing number of orthogeriatric patients. Although the proximal tibia fracture is rare in that it comprises only 1% of all fractures [4], the incidence in the elderly population is increasing [5, 6]. In contrast to a younger patient, for the orthogeriatric patient the underlying cause is mostly a low energy trauma. Due to the often preexisting osteoporosis that is present in that population, we see a lot more complex fractures with a big defect of the articular surface [7]. Because of those defects, most of the patients who undergo an open reduction and internal fixation (ORIF) will only be allowed a partial weight bearing postoperatively. But for the elderly patient the immediate postoperative mobilization with full-weight bearing is crucial to maintain the mobility they had prior to the operation. It is a known fact that if, due to reduced proprioceptive sensibilities or to preexisting comorbidities, a partial weight bearing cannot be achieved, the risk of thrombosis, pulmonary embolism, and pneumonia, as well as the reduction of muscular mass (and proprioceptive abilities), increases postoperatively. Those factors can lead to a complete loss of mobility and also to an increase rate of the overall mortality.

## 2. Methods

In this retrospective study we collected the data of 30 patients which were treated with a TKA for a proximal tibia fracture between 01/2008 and 12/2014 in our institution. This patient group was evaluated using the Knee Society Score (KSS) rating system [8] and the WOMAC score [9]. The mean follow-up was 27 months (12–48 months) with a minimum follow-up of 12 months. Overall we were able to collect the data from 73% of all patients treated with a TKA for a proximal tibia fracture in this period. 13 patients were examined in our hospital during an outpatient visit. 9 patients could only be reached via telephone due to various reasons. This latter group of patients was included in the study despite the absence of a clinical examination. The WOMAC score and the functional part of the KSS can be obtained without a physical examination of the patient. For the patients we could not examine during the follow-up, their range of motion was obtained with the help of their physiotherapist or their physician at home. Concerning the stability (anteroposterior and mediolateral) all the patients surveyed by phone had coupled/hinged prostheses models, so a normal stability was assumed. In addition to the scores we collected some statistical data of each patient: the age at the time of surgery, the AO-classification of the fracture, the comorbidities, the type of prosthesis, the average length of the operation and hospitalization, and the postoperative complications were recorded.

## 3. Results

The mean age of the patient at the time of surgery was 78.4 (59–93) years. We treated 17 female and 13 male patients. The fractures were classified according to the AO-classification. We had a total of 0 type A-fractures, 13 type B-fractures (Figure 2), and 17 type C-fractures (Figure 3) (6x AO 41B2, 5x AO 41B3, 5x AO 41C1, 7x AO 41C2, and 7x 41C3) (Table 2). The NextGen/Rotating Hinge prosthesis (Zimmer) was 13x (1x41B2, 2x41B3, 2x41C1, 3x41C2, and 5x41C3), the Innex SC prosthesis (Zimmer) was 11x (1x41B2, 2x41B3, 3x41C1, 2x41C2, and 3x41C3), and e.motion knee prosthesis (Braun) was 6x (1x41B1, 4x41B2, and 1x41B3) implanted (Table 2). In our institution the operations were only performed by two surgeons. The average length of the operation was 119 (72–150) minutes and the average length of hospitalization was 26.7 (15–35) days. *n* = 24 patients were released in a rehabilitation, *n* = 2 patients were released to a rest home, *n* = 1 patient was released to his home, and *n* = 1 patient was released in a psychiatry. *n* = 2 patients died during hospitalization, and *n* = 1 patient died 3 years later. Overall we saw 7 patients with a complication that required a further surgical treatment (wound healing deficit: 3, intraoperative periprosthetic fracture: 1, infection of the TKA: 1, residual intraarticular cement: 1, and loosening of the prosthesis component: 1). We did not see any nonsurgical complications such as a thrombosis or a pulmonary embolism. Two patients died due to complications related to a necessary further surgical treatment because of a complication of the TKA (1 prosthetic infection and 1 periprosthetic fracture) and one patient died three years after the operation due to natural causes.

**Figure 1:**
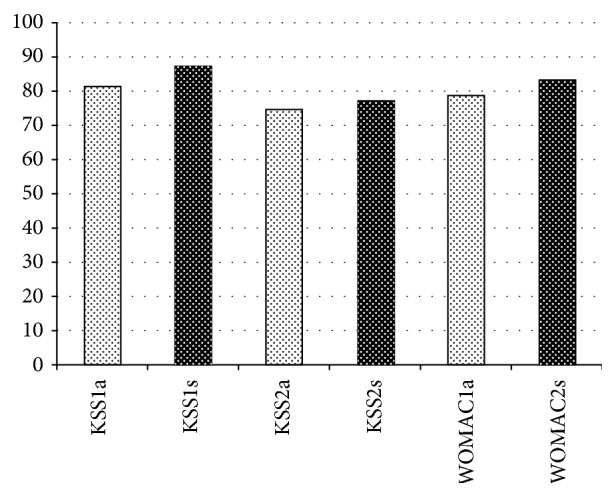
Results of all patients (a) *n* = 22 (01/2008–12/2014) versus subgroup(s) *n* = 9 (01/2013–12/2014).

**Figure 2:**
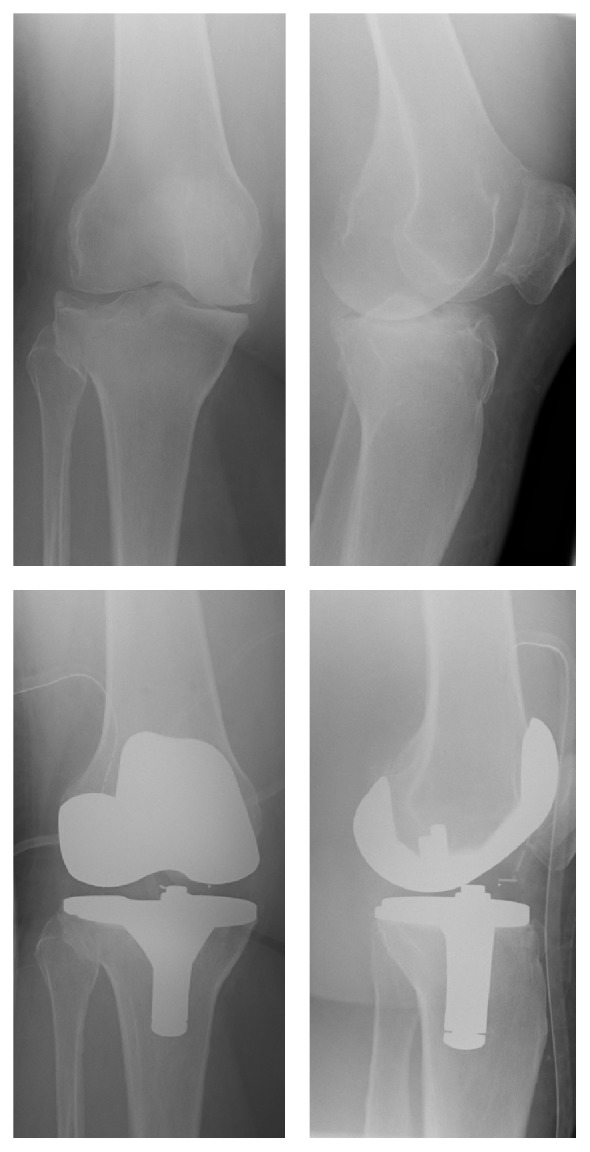
Female, age 83, tibia fracture AO 41B3, e.motion (Braun), postoperative X-ray, KSS1 83, KSS2 80, and WOMAC 87 (12th month's follow-up).

**Figure 3:**
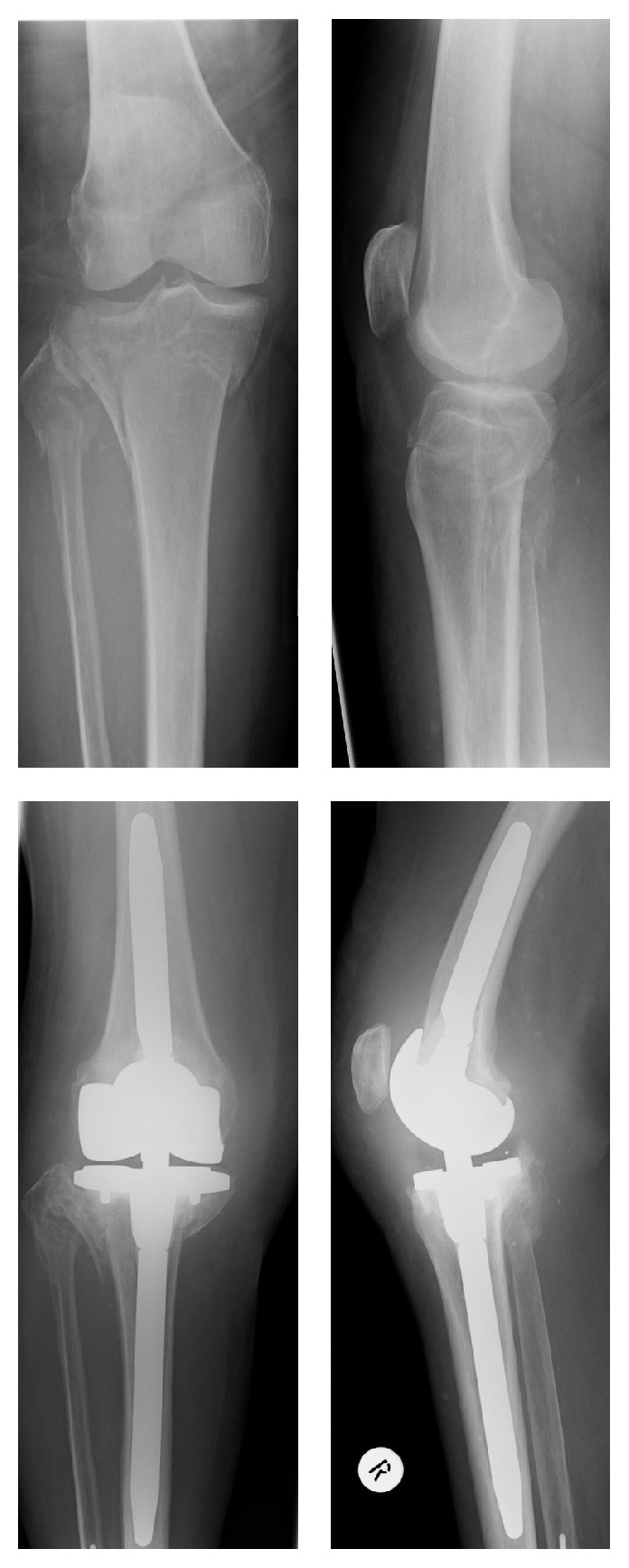
Female, age 86, tibia fracture AO 41C3, Innex SC (Zimmer), postoperative X-ray, KSS1 90, KSS2, and WOMAC 85 (13th month's follow-up).

The American Knee Society Clinical Rating Score (KSS) is composed of two components, a “knee score” (KSS1) and a “functional score” (KSS2) (80–100 points = excellent, 70–79 = good, 60–69 = fair, and below 60 = poor). In our study the patients achieved KSS1 of ø 81.1 points (94–54 points), a KSS2 of ø 74.5 (100 to −20 points), and a WOMAC score of ø 78.6 points (96.2 to 36.7 points) (Tables 3 and 4). Over the last few years we saw an improvement in the clinical outcome in our institution so we decided to set up a subgroup population who had been treated with a TKA for a proximal tibia fracture between 01/2013 and 12/2014. In the subgroup population we saw better functional results compared to the overall population group (Figure 1). These patients achieved a KSS1 of ø 87.2 points (94–69 points), a KSS2 of ø 77.2 points (100 to −20 points), and the WOMAC score of ø 83.2 points (36.7 and 97.7 points) (Tables 5 and 6).

## 4. Discussion

According to the current literature there is very limited experience with a TKA for a proximal tibia fracture. The results of our study are comparable to the good clinical results seen after a TKA shown in the literature [10–13].

There is a steady increase in the incidence of a tibia plateau fracture in the elderly over the past years [5, 6]. 95% of the patients over the age of 70 with a proximal tibia fracture have a concomitant osteoporosis [14]. Therefore in this population group we see a higher incidence of tibia plateau fracture with a significant defect of the articular surface compared to a younger population group [15]. These factors contribute to more complicated ORIFs in these patients and the literature shows poorer long-term results after ORIF in those group of patients [6, 10, 16, 17]. In cases where ORIF of an intraarticular fracture of the knee joint was preformed, there was a threefold increase in the number of cases with a reported osteoarthritis or necrosis of the tibial and femoral condyles [6]. Any secondary TKA due to a failed ORIF of a tibia plateau fracture shows poorer long-term results with an increased complication rate compared to a primary TKA [2, 10, 16, 18, 19]. Also a higher rate of secondary loss of reposition is described for the ORIF of orthogeriatric patients [6, 10, 12, 16, 20]. One of the main reasons for that is the poor bone stock in those patients due the preexisting osteoporosis. To achieve a sufficient stability for a complex proximal tibia fracture by a conventional plate osteosynthesis ORIF is often difficult [21, 22] and in most cases the osteosynthesis allows only a partial weight bearing for the first few weeks postoperatively. In our hospital we have a specialized unit for geriatric trauma patients. Our main goal is treating the joint fracture with single operation which allows a full-weight bearing postoperatively to allow early mobilization of the patient. In addition to the “surgical related” problems in this group of patients, there are age related “biological” problems which must be taken into consideration as well. Due to a sarcopenia, a preexisting limitation of mobility, cognitive impairment/dementia, or delirium, a partial weight bearing is impossible for patients in this group [4]. With the ability of a full-weight bearing postoperatively the mobilization of those patients is much easier which, in turn, may reduce the risks of medical complications [7]. In addition, the proprioceptive sensibilities and the muscle mass can be retained. The primary TKA for the treatment of a proximal tibia fracture in the elderly is a challenging operation. Over the last few years we saw better results in the clinical outcome, which we explained as being due to an improvement of the regimen within our institution, as well as an improvement in technical skills. In 80% of those fractures we used a hinged prosthesis for the TKA (NextGen (Zimmer) or Innex SC (Zimmer)). The hinged components of the prosthesis allow a high degree of stabilization for an often preexisting ligamentous instability. The longer stem components allow a better fixation in the osteoporotic bone. Those advantages mentioned above outweigh the limited options available for a revision of the prostheses if necessary. In our opinion the primary TKA for the treatment of proximal tibia fractures in the orthogeriatric patient require a very strict and individualized indication. In our study the average age was 78.4 years. Besides the age of the patient, when deciding whether to utilize a TKA there are other factors which should be taken into consideration such as the mobility prior to the injury, cognitive function, the fracture classification, the bone stock, the ligamentous stability, and the muscle mass of the patient.

## 5. Conclusion

Because of the benefits shown above for the geriatric patients, especially the immediate postoperative mobilization with full-weight bearing, associated risks like thrombosis, pulmonary embolism, and pneumonia could be reduced. Additionally the loss of the proprioceptive sensibilities and the loss of muscle mass can be minimized. And these factors, in turn, may reduce the risk of the overall mortality rate and loss of mobility in those patients. Regarding the existing literature and the good results in our study the concept of the primary total knee arthroplasty (TKA) for tibia plateau fractures in orthogeriatric patients seems to be an interesting alternative to the osteosynthetic treatment (ORIF).

We do not see this as a paradigm shift but instead as a good alternative for a certain patient group that requires a strict indication and an experienced surgeon.

## Figures and Tables

**Table 1:** Publications dealing with primary TKA according to D. Pape [4].

Publication	Year	Patients (*n*)
Kilian [10]	2003	2
Nau et al. [11]	2003	3
Nourissat et al. [12]	2006	4
Schwarz et al. [13]	2008	10
Vermeire and Scheerlinck [23].	2010	12
Malviya et al. [7]	2011	15

*n*		46

**Table 2:** AO-classification of fractures und prostheses used.

	B1	B2	B3	C1	C2	C3
Hinged prosthesis *n* = 24	0	2	4	5	5	8
e.motion *n* = 6	1	4	1	0	0	0

**Table 3:** Summary of all outcome measures 01/2008–12/2014 (*n* = 22).

	Score (max)	Mean score (range)
Knee Society Score	KSS1 (100)	81.1 (54–94)
	KSS2 (100)	74.5 (−20–100)

WOMAC score	WOMAC (100)	78.6 (36.7–96.2)

**Table 4:** Results of the Knee Society Score 01/2008–12/2014 (*n* = 22).

Knee Society Score	Excellent	Good	Fair	Poor
KSS1	14	2	1	5
KSS2	13	3	1	5

**Table 5:** Summary of all outcome measures 01/2013–12/2014 (*n* = 9).

	Score (max)	Mean score (range)
Knee Society Score	KSS1 (100)	87.2 (69–94)
	KSS2 (100)	77.2 (−20–100)

WOMAC score	WOMAC (100)	83.2 (36.7–96.2)

**Table 6:** Results of the Knee Society Score 01/2013–12/2014 (*n* = 9).

Knee Society Score	Excellent	Good	Fair	Poor
KSS1	8	0	1	0
KSS2	8	0	0	1
